# Establishing percentile charts for hip joint capsule and synovial cavity thickness in apparently healthy children

**DOI:** 10.1186/s12969-017-0136-6

**Published:** 2017-01-31

**Authors:** Zbigniew Żuber, Aleksander Owczarek, Małgorzata Sobczyk, Agata Migas-Majoch, Dorota Turowska-Heydel, Agnieszka Sternal, Justyna Michalczak, Jerzy Chudek

**Affiliations:** 1 Department of Older Children with subunits of Neurology and Rheumatology, St. Louis Regional Specialised Children’s Hospital, Krakow, Poland; 2 Department of Statistics, School of Pharmacy in Sosnowiec, Medical University of Silesia, Katowice, Poland; 3 Department of Pathophysiology, Medical Faculty in Katowice, Medical University of Silesia, Katowice, Poland

**Keywords:** Ultrasonography, Hip joint, Synovial joint space

## Abstract

**Background:**

The usefulness of musculoskeletal ultrasonography (MSUS) in paediatric population is limited by lack of reference values. One of such parameters is hip joint capsule thickness, postulated as an early measure for synovitis. However, the joint capsule is hardly a distinguished structure from slit synovial cavity in patients with little or no fluid collection. Therefore, in patients without effusion, it is more convenient to measure hip joint capsule thickness together with synovial cavity.

The aim of the study was to establish percentile chart for hip joint capsule and synovial cavity thickness (HJC&SCT) in apparently healthy children.

**Material and methods:**

The analysis included 816 US of hip joint in 408 children without musculoskeletal disorders, distributed equally throughout the whole developmental period in 18 one-year subgroups. Hip joints US was performed according to standard protocol including measurement of HJC&SCT in a single rheumatology centre by three investigators.

**Results:**

The 3rd, 10th, 25th, 50th, 75th, 90th, and 97th HJC&SCT percentile curves were depicted in the age and height charts for the combined group of girls and boys. The median HJC&SCT values were increasing with age from 3.7 (C10 - C90: 3.3 – 4.2) mm in the first year of life up to 6.7 (5.8 – 7.3) in 16 years old, and above. In a similar way the increase was seen with height from 3.9 (3.5 – 4.7) mm in shorter than 95 cm to 6.9 (6.2 – 7.4) mm in taller than 169 cm subjects. Intra-observer and inter-observer mean precision was less than 1.8 and 12.5%, respectively.

**Conclusion:**

The developed centile chart for hip joint capsule and synovial cavity thickness in the paediatric population is expected to improve detection of hip joint capsule disorders, including synovitis in juvenile idiopathic arthritis.

## Background

Musculoskeletal ultrasonography (MSUS) is a well-established relevant tool in the management of rheumatic and musculoskeletal diseases [1]. It allows evaluation of intra-articular and periarticular structures including diagnosis of synovial disease [2]. In paediatric rheumatology ultrasonography (US) can detect synovitis in patients with juvenile idiopathic arthritis (JIA) in various joints involvement (metacarpophalangeal, metatarsophalangeal, interphalangeal, wrist, ankle, knee, subtalar) and differentiated agreement with radiological imaging [3–11]. MSUS has an advantage over clinical examination in the detection of synovitis. It was shown that MSUS may also detect subclinical synovitis [7, 9, 10], and it persistent form in clinically inactive JIA [11]. Magni-Manzoni et al. showed that MSUS may detect subclinical synovitis in clinically normal joints in JIA patients [5]. They detected synovitis in 86 joints (5.5%) in 32 children with JIA performing evaluation of 52 joints [5]. Similarly, Silva et al. diagnosed subclinical synovitis using MSUS in 34.4% of children with JIA [10].

Few studies assessed hip synovitis in JIA patients applying US [7, 10, 11]. In children with JIA and active arthritis of the hip joint MSUS revealed pathological widening of the synovial joint space in 61% examinations [7]. Others reported only bone irregularities (erosions) in 4 hips, but no subclinical synovitis [11].

Collado et al. [12] in a systematic literature review focused on US as the imaging tool in the diagnosis and management of synovitis in JIA disclosing different definition used for synovitis by authors. US can evaluate synovitis at the anatomic and vascular levels. The gray-scale setting enables visualization of synovial hypertrophy and effusion, whereas the power Doppler settings allow for detecting changes in the microvascular blood flow in synovitis. The scoring system developed by the Outcome Measures in Rheumatology (OMERACT) [13] providing definitions of common pathological lesions in patients with inflammatory arthritis, cannot be applied in the paediatric population, as the normal values – percentile charts for healthy children for synovial hypertrophy were not established. Synovial membrane is hardly distinguished from the other structures of hip joint capsule and slit synovial cavity in patients with little [14] or no fluid collection, therefore we have measured the distance from outer surface of the capsule to the cartilage and name it hip joint capsule and synovial cavity thickness (HJC&SCT). Similar approach was applied by Rohrschneider at al. [15], who measured neck-capsule distance (NCD) of hip anterior recess in 166 asymptomatic children aged from 4 months to 17 years, and correlated it to growth (height).

## Aim of the study

Establishing percentile chart for hip joint capsule and synovial cavity thickness (HJC&SCT) in apparently healthy children.

## Methods

The analysis included 816 measurements of hip joint capsule thickness in 408 children without musculoskeletal disorders, distributed equally throughout the whole developmental period in 18 one-year subgroups, referred to one rheumatological centre (Department of Older Children with subunits of Neurology and  Rheumatology, St. Louis Regional Specialised Children’s Hospital in Cracow). Musculoskeletal disorders were excluded on the basis of clinical examination, laboratory findings and follow up observation.

We included patients diagnosed for increased anti-streptolysin O (ASO) titre, defective mineralization, back pain, and connective tissue diseases. Patients with symptoms of hip involvement, gait disturbances and limping, asymmetry of body (scoliosis, asymmetry of lower extremities length), and history of developmental disorders (e.g. dysplasia), injuries, transient arthritis (coxitis fugax), juvenile idiopathic arthritis (JIA), however, other rheumatic diseases were excluded.

The ultrasound (US) evaluation of the hip joints was performed according to standard protocol including measurement of hip joint capsule thickness by 3 paediatric rheumatologist trained in musculoskeletal sonography (ZZ, MS and AMM). Data were prospectively collected from July 2010 to June 2016.

The investigators obtained approval for Bioethics Committee (99/KBL/OIL/2010) for performing US measurements in paediatric patients and their use for research. Patients and parents gave informed consent for the performing of US.

### Ultrasound examination of the hip joints

The US evaluation in each patient was performed bilaterally on hip joints. Images were obtained with a Philips model HD 11 XE ultrasonography machine equipped with a 7.5-12 MHz liner transducer (accuracy 0.1 mm). A standardized procedure similar to that used by other investigators was followed, a ventral, longitudinal approach was chosen for the hip [16, 17]. Anterior longitudinal imaging is a standard procedure in our centre. During examination a patient lies on his back (supine), with hips in a neutral position, both legs straight, parallel to the ground (couch). Feet laid parallel next to each other at an angle of 90° to the ground. A linear transducer is applied parallel to the femoral neck, with proximal end located more medially (inclination angle of approx. 20°). Obtained images enable visualization of the following structures: acetabulum with cartilaginous acetabular labrum, upper extremity (head) of the femur, femoral neck, front part of the joint capsule (should be parallel to the to the femoral neck), iliofemoral ligament located above the joint capsule, iliopsoas muscle (located above the ligament), tensor fasciae late muscle (partially visible), rectus femoris muscle, and sartorius muscle (located above the iliopsoas and tensor fasciae latae muscle). The joint vascularization is visualized by Color Doppler (CD) and Power Doppler (PD).

**Fig. 1:**
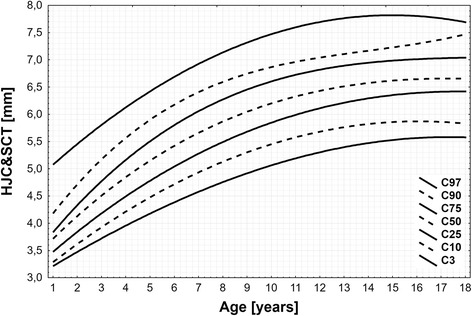
Hip joint capsule and synovial cavity thickness (HJC&SCT) centile chart for age

We measure a distance between femoral neck and joint capsule (the so-called synovial joint space, SJS) – if visible, and the distance between femoral neck and the external surface of the joint capsule - HJC&SCT (Fig. 1), as the separate measurement of SJS is difficult in subjects without significant collection of the joint fluid.

### Intra-observer variability

The inter-observer and intra-observer variability was analyzed on the basis of repeated duplicated measurements performed for 17 hip joints in 17 randomly chosen healthy children, independently by 3 sonographers. The results of previously performed measurements were blinded to the other sonographers.

### Statistical analysis

Statistical analysis was performed using STATISTICA 10.0 PL (StatSoft, Poland, Cracow). Comparison of HJC&SCT values between left and right side, for each age group, was done with the t-Student test for paired samples. For each age group all required percentiles were calculated. In the case of height 10 percentiles were calculated to divide children into the separable height-groups. Based on percentiles values the second/third order polynomials with age/height at the x axis were used to create a centile chart. Based on obtained equations all percentiles for each age/height group were then calculated and are presented in tables. In order to assess the relationship between HJC&SCT, the linear regression was used. The Cook-Weisberg test and Cameron & Trivedi’s decomposition test was used to test the residuals for heteroskedasticity as well as the violation of skewness and kurtosis assumptions in linear regression. Multicollinearity was evaluated by calculating the variance inflation factor (VIF), which should not exceed 5. As a measure of effect size for regression analysis, we used η^2^, which is the proportion of the total variance attributed to an effect. Larger values of η^2^ indicate greater influence on the dependent variable.

The intra-observer variability (mm) was calculated as a mean difference with 95% confidence interval of two following HJC&SCT measurement in a set of 34 randomly chosen patients. Intra-rater and inter-observer variability CV was calculated for this set of patients according to Chesher [18].

## Results

The analysis of hip joint capsule and synovial cavity thickness (HJC&SCT) included 816 measurements of hip joint capsule thickness in 408 apparently healthy children (254 (62,2%) girls and 154 (37,8%) boys), in 18 one-year subgroups, equally distributed through the whole of developmental period.

Similar values were observed for corresponding measurements of the left and right hip joints, all p values of the t-Student test were above 0.05. Mean difference of HJC&SCT values between left and right hip joints, for all children, was 0.02 ± 0.31 mm (95% CI: −0.01 ÷ 0.05; *p* = 0.11), and maximum difference of 0.5 mm. Therefore the analysis was performed without separation for the left and right joint measurements. In addition we showed no significant difference for girls and boys (mean difference of HJC&SCT values between groups was −0.09 mm with 95% CI: −0.25 ÷ 0.06). This justified the combine analysis for both genders.

**Fig. 2:**
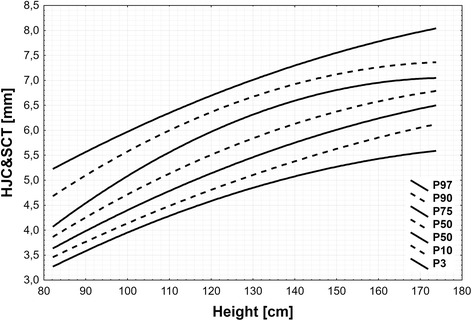
Hip joint capsule and synovial cavity thickness (HJC&SCT) centile chart for height

The median values of HJC&SCT were increasing with age from 3.7 (C10-C90: 3.3 – 4.2) mm in the first year of life up to 6.7 (5.8 – 7.3) mm in 16 years old, and then remained constant in older children (Table 1 and Fig. 1). In a similar way median values of HJC&SCT were increasing with height from 3.9 (3.5 – 4.7) mm in shorter than 95 cm to 6.9 (6.2 – 7.4) mm in taller than 169 cm (Table 1 and Fig. 2). Similar HJC&SCT values were observed for corresponding height groups of boys and girls.

**Table 1:** The values of 3rd, 10th, 25th, 50th, 75th, 90th, and 97th of hip joint capsule and synovial cavity thickness (HJC&SCT) for age and height

	Number of subjects	Percentiles						
		C97	C90	C75	C50	C25	C10	C3
Age [yrs]								
1	17	5.07	4.18	3.83	3.71	3.47	3.29	3.21
2	13	5.45	4.71	4.33	4.13	3.84	3.62	3.47
3	29	5.80	5.17	4.77	4.51	4.18	3.93	3.72
4	27	6.13	5.56	5.16	4.85	4.49	4.21	3.95
5	20	6.42	5.90	5.50	5.15	4.78	4.47	4.18
6	24	6.69	6.17	5.79	5.42	5.04	4.71	4.38
7	21	6.93	6.40	6.04	5.66	5.27	4.93	4.58
8	23	7.14	6.59	6.25	5.87	5.49	5.13	4.75
9	23	7.32	6.74	6.42	6.06	5.67	5.30	4.92
10	22	7.47	6.86	6.55	6.21	5.84	5.45	5.06
11	22	7.60	6.95	6.66	6.35	5.98	5.57	5.19
12	23	7.70	7.02	6.73	6.46	6.10	5.68	5.30
13	26	7.77	7.09	6.78	6.55	6.20	5.76	5.40
14	30	7.81	7.14	6.81	6.62	6.28	5.82	5.47
15	26	7.83	7.20	6.82	6.67	6.34	5.85	5.53
16	28	7.81	7.25	6.81	6.71	6.38	5.86	5.57
17	24	7.77	7.33	6.79	6.74	6.40	5.85	5.58
18	10	7.70	7.41	6.75	6.75	6.40	5.82	5.58
Height [cm]								
< 95	39	5.23	4.69	4.06	3.89	3.62	3.47	3.23
95–106	42	6.01	5.63	5.12	4.79	4.41	4.18	3.93
107–117	42	6.44	6.11	5.65	5.27	4.84	4.58	4.30
118–127	40	6.82	6.51	6.09	5.69	5.22	4.94	4.61
128–137	38	7.10	6.77	6.39	5.99	5.50	5.21	4.83
138–148	41	7.38	7.02	6.64	6.28	5.77	5.48	5.03
149–156	42	7.64	7.22	6.85	6.55	6.04	5.74	5.21
157–162	40	7.80	7.31	6.94	6.69	6.18	5.90	5.31
163–168	40	7.90	7.36	6.99	6.78	6.29	6.01	5.37
≥ 169	44	8.05	7.41	7.03	6.91	6.43	6.17	5.44

There was not any patient with synovial effusion, therefore the synovial joint space was not measured.

### Inter-observer and intra-observer variability

We found a high level of agreement between and within investigators. The intra-observer variability was calculated for each sonographer separately [mean difference (95% CI): 0 (−0.34 – 0.34); 0.04 (−0.29 – 0.38); −0.01 (−0.35 – 0.33) mm; corresponding to intra-rater CV: 0.5%; 0.7 and 1.8%]. The inter-observer variability CV and correlation coefficient between consecutive pairs of three corresponding sonographers were as follows: 12.3% (*r* = 0.981; *p* < 0.001), 12.4% (*r* = 0.974; *p* < 0.001) and 12.5% (*r* = 0.973; *p* < 0.001).

### Percentile chart

Percentiles for HJC&SCT were calculated for each one year age group and based on this values centile chart against age presented on Fig. 1 were created. Based on those equations all percentiles for each age group were then calculated and are presented in the Table 1. The range of centile values changes from 3.21 mm for one year-old children to 7.83 mm for fifteen year-old children.

In a similar way percentiles for HJC&SCT were calculated for 10 equinumerous, separable height groups and are presented on Fig. 2. Based on fitted equations all percentiles for each height group were then calculated and shown in Table 1. The range of centile values changes from 3.22 mm for the shortest children to 8.05 mm for the tallest children.

### Regression analyses

Linear regression coefficients were calculated for the relationship between HJC&SCT and age [for each year HJC&SCT raised by ® = 0.048 (SE(®) = 0.016; *p* < 0.01)] and height [for each centimetre of height HJC&SCT raised by ® = 0.022 (SE(®) = 0.003; *p* < 0.001)]. Height had stronger than age effect on HJC&SCT (η^2^ = 0.146 and η^2^ = 0.024; respectively). A proper usage of the regression model was confirmed by the Cameron&Trtivedi’s test (∣^2^ = 12.82; *p* = 0.118).

## Discussion

To the best of our knowledge this is the largest study that analysed the hip joint capsule thickness (measured together with slit synovial cavity) in a healthy paediatric population and the only one that created the percentile chart for age and height. Our study revealed that the hip joint capsule thickness is more related to height than age and not affected by gender. This allows to score the thickness of hip capsule in the Caucasian paediatric patients, and detect variety of synovial abnormalities, including inflammatory (synovitis) and non-inflammatory changes (eg. mucopolysaccharidosis [19, 20]), before irreversible structural changes in cartilage develop [21]. It is important from clinical point of view, as hip joints are considered difficult for clinical detection of synovitis in JIA patients [22]. While, its occurrence may change the initial diagnosis from oligoarticular to polyarticular category, and a long term prognosis.

Routinely performed X-ray imaging is not useful in the early stage of arthritis due to the lack of sensitivity for detection of synovitis and synovial effusion. Only magnetic resonance (MR) and US imaging are recommended in patients with synovial involvement. Laurell et al. demonstrated good correlation of anterior and posterior capsular distance between the US and MR in spontaneous external rotation of 10–15° and in internal rotation of 45° [23].

Early diagnosis of JIA (especially polyarticular) and introduction of the therapy with disease-modifying antirheumatic drugs (DMARDs) – usually methotrexate – the current ‘gold standard’ therapy, is improving the long-term outcome. The shorter interval between symptom onset and the early initiation of aggressive treatment regimen were associated with less joint erosions in the follow-up period [24, 25]. Involvement of hip joints, frequent in JIA children with enthesitis-related arthritis or polyarthritis subtypes, is related with worse long-term prognosis and increased risk for disability [26], and subsequent hip arthroplasty in young adults [27]. Therefore, potentially hip US with HJC&SCT measurement may be apply for detection of subclinical synovitis even without effusion for selection of patients and that can most benefit from more aggressive therapy. HJC&SCT measurement might also be useful for synovitis monitoring during the therapy. However, these hypotheses require prospective studies.

We would like to stress that HJC&SCT measurement is quite ease for performing. As previously shown, the measurement (anterior and posterior capsular distance) is slightly affected by rotation [23]. Therefore, it should be performed is one position, regardless the difference between external rotation and the neutral position was not significant [15]. A more important seems to be inter-sonographer variability. In our study, the inter-observer variability was less than 12.5% and much greater than intra-observer variability (less than 1.8%), suggesting some difference in the transducer positioning by sonographers. Therefore, optimally repeated measurements during patients follow-up in the centre, should be performed by a single sonographer.

The main limitation of our study is the lack of comparative analysis between US and another imaging modality as MR, which is believed to be the ‘gold standard’ in musculoskeletal imaging. However, such a comparison has been previously performed, showing good correlation [23]. Furthermore, the sample size of our cohort is was too small to reveal subtle differences between boys and girls in the early pubertal period. Finally, we have included Caucasians only. Consequently the presented centile chart have to be used with caution for non-Caucasian populations.

## Conclusion

The developed centile chart for hip joint capsule and synovial cavity thickness in the paediatric population is expected to improve detection of hip joint capsule disorders, including synovitis in juvenile idiopathic arthritis.

## Abbreviations

CD: Color doppler
DMARDs: Disease-modifying antirheumatic drugs
HJC&SCT: Hip joint capsule and synovial cavity thickness
JIA: Juvenile idiopathic arthritis
MRI: Magnetic resonance imaging
MSUS: Musculoskeletal ultrasonography
PD: Power doppler
US: Ultrasonography
